# Exploring and exploiting cuticle biosynthesis for abiotic and biotic stress tolerance in wheat and barley

**DOI:** 10.3389/fpls.2022.1064390

**Published:** 2022-11-10

**Authors:** Xiaoyu Wang, Cheng Chang

**Affiliations:** College of Life Sciences, Qingdao University, Qingdao, Shandong, China

**Keywords:** wheat, barley, breeding, cutin, wax, cuticle, abiotic stress, biotic stress

## Abstract

Wheat and barley are widely distributed cereal crops whose yields are adversely affected by environmental stresses such as drought, salinity, extreme temperatures, and attacks of pathogens and pests. As the interphase between aerial plant organs and their environments, hydrophobic cuticle largely consists of a cutin matrix impregnated and sealed with cuticular waxes. Increasing evidence supports that the cuticle plays a key role in plant adaptation to abiotic and biotic stresses, which could be harnessed for wheat and barley improvement. In this review, we highlighted recent advances in cuticle biosynthesis and its multifaceted roles in abiotic and biotic stress tolerance of wheat and barley. Current strategies, challenges, and future perspectives on manipulating cuticle biosynthesis for abiotic and biotic stress tolerance in wheat and barley are discussed.

## Introduction

As the first plants domesticated about 10,000 years ago, wheat and barley are important cereal crops used extensively for human food and animal feed (Haas et al., 2019; Levy and Feldman, 2022). The global population is projected to reach 9.7 billion by 2050 and rise further to 11.2 billion in 2100, which drives the demand for wheat and barley grains (Lee, 2011). However, yields and quality of wheat and barley are adversely affected by numerous environmental stresses such as water deficit (drought), high salinity, extreme temperatures (heat and cold), and attacks of pathogens and pests (P&Ps) (Hura, 2020). For instance, drought stress was documented to reduce yields of 50%-90% and 49%-87% in drought-susceptible cultivars of wheat and barley respectively (Samarah, 2005; Daryanto et al., 2016). Soil salinity affects about 20% of global cultivated land, and seriously threatens the growth and production of glycophytes wheat and barley (Zörb et al., 2019). Temperature stresses such as chilling, freezing, and heat have become more frequent due to climate change and reduced grain yields and quality of wheat and barley (Jacott and Boden, 2020). In addition, a plethora of P&Ps, including pathogenic fungi, oomycetes, bacteria, viruses, nematodes, and herbivorous insects, were responsible for above 20% yield loss in wheat and barley (Savary et al., 2019). Developing and cultivating resistant varieties of wheat and barley are, therefore, essential for ensuring food security under environmental challenges.

As the outmost surface of terrestrial plants, lipophilic cuticle predominantly covers plant aerial organs like non-woody stems, leaves, flowers, and fruits, and protects plant tissues from abiotic and biotic stresses such as drought, salinity, heat, cold, ultraviolet (UV) radiation, mechanical damages, and P&Ps attacks (Domínguez et al., 2017; Kong et al., 2020a; Li and Chang, 2021). In addition to these protective roles, the cuticle also regulates plant developmental processes by inhibiting organ fusion and promoting lateral root formation (Kurdyukov et al., 2006; Ingram and Nawrath, 2017; Berhin et al., 2019). It has been demonstrated that the expression of cuticle biosynthesis genes is governed by DNA-binding transcription factors (TFs), mediators, and epigenetic regulators (Lee and Suh, 2015; Lee and Suh, 2022). At the same time, there is increasing evidence that cuticle biosynthesis mechanisms could be exploited for crop improvement (Wang et al., 2020; Liu et al., 2022b). Although past decades have seen a great advance in the understanding mechanisms of plant cuticle biosynthesis, most of this progress was achieved in model plants. Herein, we focus on recent studies exploring the mechanism of cuticle biosynthesis and its roles in the tolerance of wheat and barley to biotic and abiotic stresses. Potentials, strategies, challenges, as well as future perspectives on harnessing cuticle biosynthesis to improve abiotic and biotic stress tolerance in wheat and barley are discussed.

## Cuticle composition and biosynthetic machinery in wheat and barley

As a hydrophobic layer covering the plant aerial epidermis, the cuticle is generally composed of lipid, phenolic, and polysaccharide compounds, and its hydrophobic property is mainly conferred by the lipid components cutin and wax (Reynoud et al., 2021). Cutin largely consists of cross-linked polyester of oxygenated C16 and C18 fatty acids, as well as their derivatives, whereas cuticular wax mixtures contain very-long-chain (VLC, >C20) fatty acids, alkanes, aldehydes, alcohols, esters, and ketones (Bhanot et al., 2021). Although lipophilic wax and cutin constitute the major components of plant cuticle, the composition of wax and cutin varies among plant species, organs, developmental stages, and environmental conditions. For instance, VLC alkanes are the major wax constituents of seedling leaves and stems in *Arabidopsis*, whereas VLC alcohols dominate the wax compositions of seedling leaves in wheat and barley (Rowland et al., 2006; Wang et al., 2015; Li et al., 2018). Notably, *Arabidopsis* does not produce β-diketones that are abundant in the cuticles covering spikes, flag leaves and stems at flowering wheat and barley plants (Hen-Avivi et al., 2016; Schneider et al., 2016). In addition, C18:0 18-OH acids are identified as the major cutin monomers in the seedling leaves of wheat and barley, whereas C18:2 diacids dominate the cutin composition of *Arabidopsis* seedling leaves (Hong et al., 2017; Kong and Chang, 2018; Li et al., 2018).

In plant epidermal cells, biosyntheses of cutin and wax occur in the endoplasmic reticulum (ER) through the modification of C16 and C18 fatty acids trafficked from the plastid (Yeats and Rose, 2013). For the cutin biosynthesis, C16 and C18 fatty acids sequentially undergo esterification, aliphatic chain elongation, hydroxylation, and acyltransferation to synthesize the cutin precursor *sn-*2 monoacylglycerols (2-MAGs) (Fich et al., 2016; Philippe et al., 2020). Long-chain acyl-coenzyme A synthases (LACS) catalyze the esterification of C16 and C18 fatty acids with coenzyme A (CoA) (Fich et al., 2016; Philippe et al., 2020). Cytochrome P450 enzymes (CYP77 and CYP86) and epoxide hydrolases (EH) mediate the hydroxylation of acyl-CoAs, and then glycerol-3-phosphate acyltransferase (GPAT) enzymes convert acyl-CoAs to 2-MAGs precursors (Lee et al., 2020). Cutin precursors are then exported out of plant cell *via* plasma membrane (PM) localized ATP binding cassette transporter subfamily G (ABCG) proteins and deposited into the cuticle, where cutin synthase (CUS) enzymes catalyze the cutin polymerization (Hong et al., 2017; Elejalde-Palmett et al., 2021; Philippe et al., 2022).

For the wax biosynthesis, fatty acid elongase (FAE) enzyme complexes comprising ketoacyl-CoA synthases (KCS), ketoacyl-CoA reductases (KCR), hydroxyacyl-CoA dehydratases (HCD), and enoyl-CoA reductases (ECR) function together with ECERIFERUM2-LIKE (CER2-LIKE) proteins to catalyze the aliphatic chain elongation of C16 and C18 acyl-CoAs, leading to the formation of VLC acyl-CoAs (Haslam et al., 2015; Haslam and Kunst, 2021; Kim J. et al., 2022). VLC acyl-CoAs could be converted to VLC alkanes by a VLC alkane-forming complex consisting of ECERIFERUM1 (CER1), CER1-LIKE1, CER3, and cytochrome B5 (CYTB5) proteins (Pascal et al., 2019). VLC alkanes then undergo hydroxylation mediated by the CYP95A family cytochrome P450 enzyme midchain alkane hydroxylase 1 (MAH1) to form VLC secondary alcohols and ketones in the alkane-forming pathway (Greer et al., 2007). As an alternative direction, VLC acyl-CoAs could enter the alcohol-forming pathway and are converted to VLC primary alcohols by fatty acyl-coenzyme A reductase (FAR) CER4 and acyl-CoA desaturase LIKE4 (ADS4/CER17) (Rowland et al., 2006; Yang et al., 2017). VLC primary alcohols and acyl-CoAs serve as precursors in the subsequent biosynthesis of wax esters catalyzed by a bifunctional wax ester synthase/diacylglycerol acyltransferase WSD1 (Li et al., 2008; Patwari et al., 2019). These wax constituents such as VLC fatty acids, aldehydes, alkanes, alcohols, ketones, and esters are transported through the Golgi and trans-Golgi network (TGN)-trafficking pathways to the PM, and then exported to the cuticle *via* ABCG subfamily half transporters and the lipid transfer proteins (LTPs) (Pighin et al., 2004; Debono et al., 2009; Ichino and Yazaki, 2022).

Although most of these advances in the understanding of cuticle biosynthesis were derived from studies in the model plants like *Arabidopsis* and tomato, evolutionarily conserved functions are widely displayed by cuticle biosynthesis genes of wheat and barley. On one hand, ectopic expression of cuticle biosynthesis genes derived from wheat and barley could significantly enhance cuticle formation in *Arabidopsis*. Indeed, overexpression of wheat *TaCER1-1A* in *Arabidopsis* could enhance stem and leaf accumulation of cuticular wax (Li et al., 2019). Heterologous expression of wheat *TaFAR2*, *TaFAR3*, and *TaFAR4* in *Arabidopsis cer4-3* mutant defective in the production of C24 and C26 primary alcohols results in the increased accumulation of primary alcohols (Wang et al., 2016). On the other hand, knockdown or knockout of wheat and barley genes orthologous to *Arabidopsis* cuticle biosynthesis genes usually attenuated plant cuticle biosynthesis. Silencing of wheat *TaECR* and *TaKCS6 via* virus-induced gene silencing (VIGS) led to reduced wax accumulation in wheat leaves (Wang et al., 2019; Kong et al., 2020b). Likewise, barley mutant enhanced *Magnaporthe resistance gene1* (*emr1*) carrying a mutation in *HvKCS6* is depleted of leaf wax (Weidenbach et al., 2014). Although some cuticle biosynthesis genes exhibited functional conservation among *Arabidopsis*, wheat, and barley, functional divergence is also observed in some cuticle biosynthesis genes. For instance, *Arabidopsis* T-DNA tagged mutant *kcs1-1* displayed a marginal change in the total wax load compared with the wild-type plants. In contrast, the barley *eceriferum-zh* (*cer-zh*) mutant carrying mutation in the *KCS1* gene exhibited significantly reduced wax accumulation (Todd et al., 1999; Li et al., 2018). These studies support the idea that, although cuticle biosynthetic machinery is highly conserved among model and crop plants, the functional divergence has been acquired by some cuticle biosynthesis genes in wheat and barley.

As oxidized hydrocarbons, β-diketones are cuticular wax components of wheat and barley rather than the model plant *Arabidopsis* (von Wettstein-Knowles, 1972; Kosma and Rowland, 2016; von Wettstein-Knowles, 2017). Characterization of barley *eceriferum-c* (*cer-c*), *cer-q*, and *cer-u* mutants with altered glaucousness traits shed novel light into the biosynthesis of β-diketone (Kosma and Rowland, 2016; von Wettstein-Knowles, 2017). Map based cloning revealed that barley *Cer-c*, *Cer-q* and *Cer-u* genes reside in the 101 kb *Cer-cqu* gene cluster as the order *Cer-c*, *Cer-u*, *Cer-q*, and encode a chalcone-synthase-like diketone synthase (DKS), a putative lipase/carboxyl transferase, and a cytochrome P450 hydroxylase, respectively (Schneider et al., 2016). As extensively discussed by von Wettstein-Knowles, β-diketones are proposed to be synthesized from C12, C14, C16 fatty acid and C16-CoA *via* a polyketide-like pathway involving CER-C, CER-Q, CER-U together with components of FAE complex (von Wettstein-Knowles, 2017). Transcriptomic analysis using chromosomearm substitution lines (CASLs) of wild emmer together with the subsequent gene silencing assays revealed that wheat *W1* locus contains a similar gene cluster harboring *Diketone Metabolism-PKS* (*DMP*), -*Hydrolase* (*DMH*), and -*CYP450* (*DMC*) genes essential for β-diketone biosynthesis, suggesting that the conserved metabolic gene cluster mediates β-diketone biosynthesis in wheat and barley (Hen-Avivi et al., 2016).

## Regulatory mechanisms of cuticle biosynthesis in wheat and barley

Accumulating evidence support that the expression of cuticle biosynthesis genes in wheat and barley is tightly governed by TFs, mediators, and epigenetic regulators. SHINE (SHN) clade of AP2 domain TF AtSHN1 and its close homologs AtSHN2 and AtSHN3 were firstly identified as transcriptional activators of cuticle lipid biosynthesis in *Arabidopsis* (Aharoni et al., 2004; Broun et al., 2004; Kannangara et al., 2007). Barley TF WAX INDUCER1 (HvSHN1/WIN1) and wheat TF TaSHN1/WIN1 are homologs of *Arabidopsis* SHN1. Knockdown of *HvSHN1/WIN1* by VIGS resulted in the reduced accumulation of cuticular lipid in barley spikelets, whereas ectopic expression of *HvSHN1/WIN1* in tobacco could activate the expression of wax biosynthesis gene *NtCER1* and resulted in the altered cuticle property (Kumar et al., 2016; Djemal and Khoudi, 2021). Likewise, Knockout or knockdown of *TaSHN1/WIN1* expression in bread wheat attenuated wax and cutin biosynthesis, whereas overexpression of *TaSHN1/WIN1* led to enhanced wax accumulation in transgenic wheat plants (Bi et al., 2018; Kong and Chang, 2018). This evidence supports that TFs TaSHN1/WIN1 and HvSHN1/WIN1, resembling their counterparts in *Arabidopsis*, positively regulate cuticle biosynthesis in wheat and barley. PpWIN1, an SHN1 homolog in *Physcomitrium patens*, was recently revealed to stimulate cuticle formation in *P. patens* and *Arabidopsis* by activating cutin and wax biosynthesis genes, suggesting that the transcriptional activation of cuticle biosynthesis by TF SHN1/WIN1 might be conserved from moss to higher land plants including wheat and barley (Kim R. J. et al., 2022).

In addition to SHN/WINs, myeloblastosis (MYB) and basic helix-loop-helix protein (bHLH) type TFs are identified as key regulators of wheat cuticle biosynthesis. For instance, the wheat MYB TF TaMYB74 was revealed to transactivate wheat cuticle biosynthesis-related gene *TaSHN1/WIN1* and respond to drought stress (Bi et al., 2016). Another wheat MYB TF TaEPBM1 could directly bind to the promoter region of the wax biosynthesis gene *TaECR* and activate *TaECR* expression (Kong et al., 2020b). TaMYB96, allelic to TaEPBM1, was recently demonstrated to target *TaCER1-6A*, *TaCER1-1A*, and *TaFAR4*, and positively regulate wax biosynthesis as well, supporting that TaMYB96/TaEPBM1 potentiates wheat cuticle biosynthesis *via* directly activating wax biosynthesis genes (He et al., 2022). In *Arabidopsis*, bHLH TFs CFLAP1 and CFLAP2 were demonstrated to negatively regulate wax biosynthesis and cuticle formation (Li et al., 2016). The wheat bHLH TF TaKPAB1 was demonstrated to recognize the E-box cis-element in the promoter of wax biosynthesis gene *TaKCS6* and activate the transcription of *TaKCS6* (Wang et al., 2019). Knockdown of *TaKPAB1* and *TaKCS6* expression by VIGS results in reduced wax accumulation, suggesting that transactivation of *TaKCS6* by TaKPAB1 positively regulates wheat cuticular wax biosynthesis (Wang et al., 2019).

As an essential component of a highly conserved mediator complex, CYCLIN-DEPENDENT KINASE8 (CDK8) functions as a transcriptional co-regulator to activate or repress transcription of target genes (Mao et al., 2019; Agrawal et al., 2021; Chen J. et al., 2022). In *Arabidopsis*, CDK8 is physically associated with the TF AtSHN1 and positively regulates plant cuticle formation (Zhu et al., 2014). TaCDK8, the wheat homolog of Arabidopsis AtCDK8, also interacts with TaSHN1/WIN1 and activates wax and cutin biosynthesis in bread wheat, suggesting that positive regulation of cuticle biosynthesis by CDK8-WIN1 module might be conserved among dicots and monocots (Kong and Chang, 2018). Notably, TaCDK8 could directly phosphorylate TaSHN1/WIN1, which is essential to the transcriptional activation mediated by TaSHN1/WIN1 (Kong and Chang, 2018). Silencing of *TaCDK8* and *TaWIN1* by VIGS attenuated wheat wax biosynthesis, further supporting the idea that mediator subunit TaCDK8 phosphorylates the TF TaSHN1/WIN1 to stimulate the expression of wheat biosynthesis genes (Kong and Chang, 2018).

Histone modification and chromatin remodeling are considered important epigenetic mechanisms in the regulation of plant development and environmental adaptation (Chang et al., 2020; Kumar et al., 2021; Zhi and Chang, 2021). Arabidopsis histone methyl transferases SET DOMAIN GROUP8 (AtSDG8), AtSDG25, histone E3 ligases HISTONE MONOUBIQUITINATION 1 (AtHUB1), AtHUB2, and histone acetyltransferase GENERAL CONTROL NON-REPRESSED PROTEIN5 (AtGCN5) play important roles in the regulation of cuticle biosynthesis (Ménard et al., 2014; Lee et al., 2016; Wang et al., 2018). It was demonstrated that wheat TF TaEPBM1 could directly bind to the transcriptional coactivator TaADA2, an interacting partner of TaGCN5 in the histone acetyltransferase module of Spt-Ada-Gcn5 Acetyltransferase (SAGA) complex (Kong et al., 2020b). *Via* association with TaEPBM1, the TaGCN5-TaADA2 module is recruited to the *TaECR* promoter region to mediate histone acetylation (Kong et al., 2020b). Silencing of *TaGCN5* and *TaADA2* by VIGS resulted in reduced *TaECR* expression and decreased wax accumulation, suggesting that epigenetic activation of *TaECR* by histone acetyltransferase complex TaGCN5-TaADA2 triggers wheat wax biosynthesis (Kong et al., 2020b). Likewise, the CHD3-type chromatin remodeling factor TaCHR729 interacts with TF TaKPAB1 and is recruited to the promoter region of *TaKCS6* (Wang et al., 2019). Through mediating deposition of permissive epigenetic mark H3K4me3, TaCHR729 promotes *TaKCS6* expression and positively regulates wheat wax biosynthesis (Wang et al., 2019). These studies provide evidence that the expression of the wheat cuticle biosynthesis gene is epigenetically governed by multiple epigenetic regulators, including histone modifying enzymes and chromatin remodeling factors.

Interplays of cuticle biosynthesis with other epidermal developmental processes like trichome formation have been extensively discussed by prior reviews (Ingram and Nawrath, 2017; Berhin et al., 2022). Cuticle functions in concert with stomata to tightly control water and gas exchange essential for plant photosynthesis and environmental adaptation. Molecular characterization of barley *cer-g.10* and *cer-s.31* mutants exhibiting wax-deficiency and stomatal misarrangement demonstrated that *Cer-g* and *Cer-s* genes respectively encode a YODA-like (YDA) MAPKKK HvYDA1 and a BREVIS-RADIX (BRX) domain protein HvBRX-Solo, two signaling components in stomatal development (Liu et al., 2022a). Epidermal phenotype analysis of *cer-g.10*, *cer-s.31* and *cer-g.10 cer-s.31* double mutants revealed that HvYDA1 and HvBRX function in a common pathway to control wax deposition and epidermal patterning (Liu et al., 2022a). RNA sequencing (RNA-seq) analysis showed that HvYDA1 and HvBRX-Solo coregulate downstream genes associated with cuticle development, epidermal differentiation and patterning, further confirming that HvYDA1-HvBRX-Solo signaling module orchestrates cuticle biosynthesis and epidermal patterning in barley (Liu et al., 2022a).

## Regulation of plant abiotic stress tolerance by cuticle biosynthesis in wheat and barley

**Figure 1:**
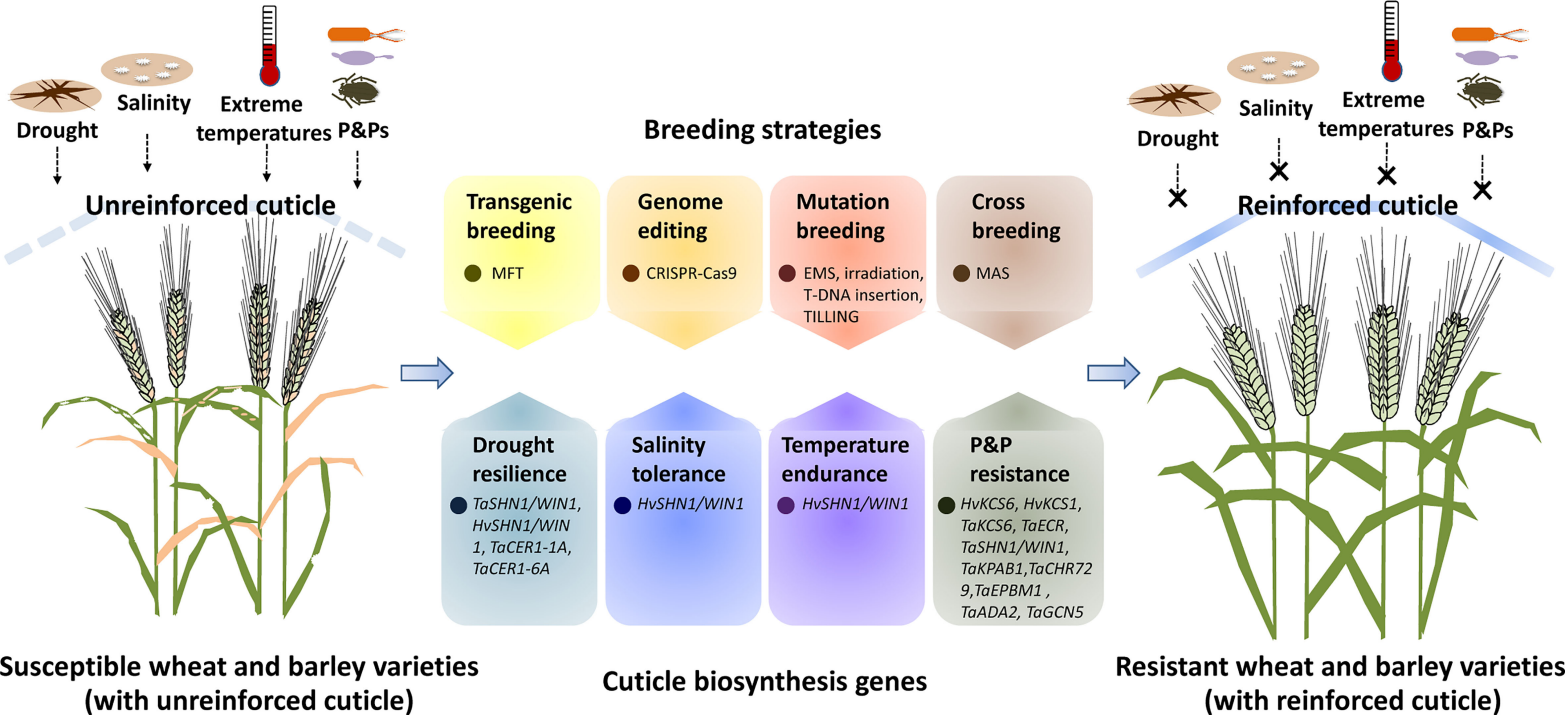
A simplified model for manipulating cuticle biosynthesis to improve abiotic and biotic stress tolerance in wheat and barley. Cuticle biosynthesis genes in wheat and barley get involved in the regulation of plant response to abiotic and biotic stresses such as drought, salinity, extreme temperatures, and attacks of pathogens and pests (P&Ps). Genetic manipulation of these cuticle biosynthesis genes by transgenic breeding, genome editing, mutagenesis breeding and cross breeding could reinforce the cuticle, resulting in improved abiotic and biotic stress resistance in wheat and barley.

By limiting non-stomatal water loss, cuticle contributes to plant adaptation to drought conditions, which has been extensively discussed in previous reviews (Xue et al., 2017; Lewandowska et al., 2020; Liu et al., 2022b). Upregulation of cuticle biosynthesis-related genes such as *CER1s*, *FARs*, *SHN1*, *MYB74*, *WXPLs* in response to drought stress has been reported in wheat and barley (Wang et al., 2015; Bi et al., 2016; Wang M. et al., 2016; Bi et al., 2017; Bi et al., 2018; Chai et al., 2018; Li et al., 2019; He et al., 2022). As summarized in **Table 1** and **Figure 1**, over-expression of these cuticle biosynthesis genes usually results in reinforced cuticle formation and enhanced plant drought tolerance. In contrast, knockout or knockdown of these cuticle biosynthesis genes expression could attenuate cuticle formation and compromise plant drought resilience. For instance, over-expression of *TaSHN1/WIN1* and *TaCER1-6A* in transgenic wheat plants resulted in enhanced wax accumulation and increase drought tolerance (**Table 1**, **Figure 1**) (Bi et al., 2018; He et al., 2022). Ectopic expression of *TaCER1-1A* and *HvSHN1/WIN1* in rice and tobacco could also alter the cuticle property and lead to enhanced plant drought resistance (**Table 1**, **Figure 1**) (Li et al., 2019; Djemal and Khoudi, 2021). Consistent with this, wheat *TaCER1-6A* knockout lines generated by CRISPR/Cas9 genome editing system displayed enhanced cuticle permeability and attenuated plant drought resilience (**Table 1**, **Figure 1**) (He et al., 2022). Reduced water retention capacity was also observed in the leaves of barley cuticle mutant *eceriferum-ym* (*cer-ym*), *cer-zv* and *eibi1* (Chen et al., 2004; Chen et al., 2011; Li et al., 2015; Li et al., 2017).

**Table 1:** Summary of cuticle biosynthesis genes contributing to abiotic and biotic stress tolerance in wheat and barley.

Type of environmental stress	Gene name	Gene product	Crop specie	Gene product family	Function of gene product	Evidence of gene function in plant resistance to environmental stress	Reference
Drought	*TaSHN1/WIN1*	TaSHN1/WIN1	*Triticum aestivum*	SHN clade of AP2 domain TF	Transcriptional activation of wax and cutin biosynthesis genes	Over-expression of *TaSHN1/WIN1* in transgenic wheat plants resulted in enhanced wax accumulation and increase drought tolerance.	(Bi et al., 2018)
	*TaCER1-6A*	TaCER1-6A	*T. aestivum*	VLC-aldehyde decarbonylase putative	Biosynthesis of VLC alkanes	Over-expression of *TaCER1-6A* in transgenic wheat plants enhanced wax biosynthesis and increased drought tolerance, whereas knockout of wheat TaCER1-6A by CRISPR/Cas9 genome editing system attenuated cuticle biosynthesis and compromised plant drought resilience.	(He et al., 2022)
	*TaCER1-1A*	TaCER1-1A	*T. aestivum*	VLC-aldehyde decarbonylase putative	Biosynthesis of VLC alkanes	Ectopic expression of *TaCER1-1A* in rice leads to enhanced plant drought resistance	(Li et al., 2019)
	*HvSHN1/WIN1*	HvSHN1/WIN1	*Hordeum vulgare*	SHN clade of AP2 domain TF	Transcriptional activation of wax and cutin biosynthesis genes	Ectopic expression of *HvSHN1/WIN1* in tobacco could alter the cuticle property and lead to enhanced plant drought resistance.	(Djemal and Khoudi, 2021)
Salinity	*HvSHN1/WIN1*	HvSHN1/WIN1	*Hordeum vulgare*	SHN clade of AP2 domain TF	Transcriptional activation of wax and cutin biosynthesis genes	Ectopic expression of *HvSHN1/WIN1* in tobacco resulted in enhanced plant resilience to salt stress.	(Djemal and Khoudi, 2021)
Extreme temperatures	*HvSHN1/WIN1*	HvSHN1/WIN1	*Hordeum vulgare*	SHN clade of AP2 domain TF	Transcriptional activation of wax and cutin biosynthesis genes	Ectopic expression of *HvSHN1/WIN1* in tobacco led to increased plant tolerance to heat stress.	(Djemal and Khoudi, 2021)
P&P attacks	*HvKCS6*	HvKCS6	*H. vulgare*	3-Ketoacyl-CoA synthase	Biosynthesis of VLC acyl-CoAs	*Bgh* conidial germination is attenuated on the barley wax biosynthetic mutant *emr1* carrying a mutation in *HvKCS6.*	(Weidenbach et al., 2014)
	*HvKCS1*	HvKCS1	*H. vulgare*	3-Ketoacyl-CoA synthase	Biosynthesis of VLC acyl-CoAs	*Bgh* conidial germination is attenuated on the barley wax biosynthetic mutant *cer-zh* carrying a mutation in *HvKCS1.*	(Li et al., 2018)
	*TaSHN1/WIN1*	TaSHN1/WIN1	*T. aestivum*	SHN clade of AP2 domain TF	Transcriptional activation of wax and cutin biosynthesis genes	Silencing of wheat *TaSHN1/WIN1* by VIGS led to attenuated cuticle biosynthesis and compromised *Bgt* germination.	(Kong and Chang, 2018)
	*TaCDK8*	TaCDK8	*T. aestivum*	Kinase component of mediator complex	Transcriptional activation of wax and cutin biosynthesis genes	Silencing of wheat *TaCDK8* by VIGS led to attenuated wax and cutin accumulation, as well as compromised *Bgt* germination.	(Kong and Chang, 2018)
	*TaKCS6*	TaKCS6	*T. aestivum*	3-Ketoacyl-CoA synthase	Biosynthesis of VLC acyl-CoAs	Silencing of *TaKCS6* by VIGS led to attenuated wheat wax accumulation and compromised *Bgt* germination.	(Wang et al., 2019)
	*TaKPAB1*	TaKPAB1	*T. aestivum*	bHLH type TFs	Transcriptional activation of wax biosynthesis genes	Knockdown of *TaKPAB1* expression by VIGS led to attenuated wheat wax accumulation and compromised *Bgt* germination.	(Wang et al., 2019)
	*TaCHR729*	TaCHR729	*T. aestivum*	CHD3 type chromatin remodeling factor	Epigenetic activation of wax biosynthesis genes	Silencing of *TaCHR729* by VIGS led to attenuated wheat wax accumulation and compromised *Bgt* germination.	(Wang et al., 2019)
	*TaECR*	TaECR	*T. aestivum*	Enoyl-CoA reductase	Biosynthesis of VLC acyl-CoAs	VIGS of *TaECR* resulted in attenuated wheat wax accumulation and compromised *Bgt* germination.	(Kong et al., 2020b)
	*TaEPBM1*	TaEPBM1	*T. aestivum*	bHLH type TF	Transcriptional activation of wax biosynthesis genes	Knockdown of *TaEPBM1* expression by VIGS led to attenuated wheat wax accumulation and compromised *Bgt* germination.	(Kong et al., 2020b)
	*TaADA2*	TaADA2	*T. aestivum*	Transcriptional coactivator	Transcriptional activation of wax biosynthesis genes	Silencing of *TaADA2* by VIGS led to reduced wheat wax accumulation and compromised *Bgt* germination.	(Kong et al., 2020b)
	*TaGCN5*	TaGCN5	*T. aestivum*	Histone acetyltransferase	Epigenetic activation of wax biosynthesis genes	VIGS of *TaGCN5* resulted in attenuated wheat wax accumulation and decreased *Bgt* germination.	(Kong et al., 2020b)
	*HvSHN1/WIN1*	HvSHN1/WIN1	*H. vulgare*	SHN clade of AP2 domain TF	Transcriptional activation of wax and cutin biosynthesis genes	Knockdown of *HvSHN1/WIN1* expression by VIGS in resistant barley cultivar resulted in reduced cuticular lipid accumulation and attenuated FHB resistance.	(Kumar et al., 2016)

As a protective shield covering aerial organs, the cuticle also protects plant tissues from other environmental stresses such as salinity, heat, cold, and UV radiation, which has been supported by a broad range of research on model and crop plants (Zhang et al., 2020; Abdullah et al., 2021; Busta et al., 2021; Benítez et al., 2022; González Moreno et al., 2022; Kan et al., 2022; Liu et al., 2022). Expression of eight wheat *FAR* genes (*TaFAR1*, *TaFAR2*, *TaFAR3*, *TaFAR4*, *TaFAR5*, *TaFAR6*, *TaFAR7*, and *TaFAR8*) are up-regulated by cold stress, and three *FAR* genes (*TaFAR2*, *TaFAR3*, and *TaFAR4*) are induced by salinity stress, suggesting that these cuticle biosynthesis *FAR* genes might get involved in wheat response to temperature and salt stress (Wang et al., 2015; Wang et al., 2016; Chai et al., 2018). Interestingly, heterologous expression of barley *HvSHN1/WIN1* gene in tobacco led to enhanced plant tolerance to heat, salt, and drought stress, further supporting the contribution of cuticle biosynthesis to abiotic stress resilience in wheat and barley (**Table 1**, **Figure 1**) (Djemal and Khoudi, 2021).

## Regulation of plant-P&P interactions by cuticle biosynthesis in wheat and barley

As the first contact interphase between aerial plant organs and P&Ps, the cuticle regulates multiple processes such as pathogen (pre)penetration, plant immunity, and pest behaviors in plant-P&P interactions, which has been summarized by previous reviews (Wang et al., 2020; Arya et al., 2021; Li et al., 2022). Accumulating evidence revealed that plant cuticle governs the interactions of P&Ps with wheat and barley. Firstly, cuticular wax signals are essential for triggering the (pre)penetration development of pathogenic powdery mildew fungi in wheat and barley. Conidial germination of the barley powdery mildew fungus *Blumeria graminis* f. sp. *hordei* (*Bgh*) is attenuated on the barley wax biosynthetic mutant *emr1* and *cer-zh* (**Table 1**, **Figure 1**) (Weidenbach et al., 2014; Li et al., 2018). Likewise, silencing of wheat wax biosynthesis genes *TaKCS6*, *TaECR*, *TaSHN1/WIN1*, *TaCDK8*, *TaKPAB1*, *TaEPBM1*, *TaADA2*, and *TaGCN5* all led to the attenuated wax accumulation and compromised germination of the wheat powdery mildew fungus *Blumeria graminis* f. sp. *tritici* (*Bgt*) (**Table 1**, **Figure 1**) (Kong and Chang, 2018; Wang et al., 2019; Kong et al., 2020b). Notably, *in vitro* application of wax constituents, VLC aldehydes, stimulate the *Bgh* germination in a dose-dependent manner and could fully restore the *Bgt* germination penalty on the wheat wax biosynthetic mutant, suggesting that VLC aldehydes are the plant wax signals essential for stimulating (pre)penetration development of *Blumeria graminis* in wheat and barley (Hansjakob et al., 2010; Kong and Chang, 2018; Wang et al., 2019; Kong et al., 2020b). Secondly, cuticle biosynthesis contributes to the immunity of wheat and barley against some pathogens. For instance, barley resistance to Fusarium head blight (FHB) is correlated with the expressions of cuticle biosynthesis genes *HvKAS2*, *HvCYP86A2*, *HvCYP89A2*, *HvLACS2* and *HvSHN1/WIN1* in resistant cultivar (**Table 1**, **Figure 1**) (Kumar et al., 2016). Knockdown of *HvSHN1/WIN1* expression by VIGS in this resistant barley cultivar resulted in reduced cuticle lipid accumulation and attenuated FHB resistance, further supporting the contribution of cuticle lipid biosynthesis to barley FHB resistance (Kumar et al., 2016). Thirdly, cuticle biosynthesis gets involved in wheat response to pest infestation. Kosma et al. reported that infestation of the pest Hessian fly leads to the up-regulation of cuticle biosynthesis genes such as *TaCER3*, *TaCER1*, *TaCER4*, *TaKCS1*, *TaKCS6*, *TaCER5*, together with the alteration in wheat wax and cutin compositions (Kosma et al., 2010). Notably, these transcriptomic and metabolic responses displayed the difference in resistant and susceptible wheat cultivars, implying that cuticle biosynthesis might play a key role in the regulation of wheat resistance against Hessian fly infestation (Kosma et al., 2010).

## Strategies, limitations, and perspectives on exploiting cuticle biosynthesis for wheat and barley improvement

As a hydrophobic shield covering aerial plant organs, the cuticle contributes to plant adaptation to environmental stresses such as drought, salinity, extreme temperatures, and P&P attacks (Arya et al., 2021; Liu et al., 2022). Through analyzing leaf wax alkane and grain yield traits in five wheat cultivars released during the past six decades, Liu et al. observed a tendency to increase and a strong correlation in leaf wax alkane concentration and grain yield across the historical wheat varieties, suggesting that increased leaf wax alkane concentration has been selected in breeding efforts for wheat production improvement (Liu et al., 2019). Exploiting cuticle biosynthesis by advanced breeding strategies such as transgenic breeding, genome editing, mutation breeding, and cross breeding could provide more avenues for wheat and barley improvement (Liu et al., 2019).

Genetic engineering of cuticle biosynthesis genes could confer plant stress resistance. For instance, the over-expression of *TaSHN1/WIN1* and *TaCER1-6A* led to wax over-accumulation and increased drought resilience in bread wheat (**Table 1**, **Figure 1**) (Bi et al., 2018; He et al., 2022). Notably, ectopic expression of *Arabidopsis AtMYB96* and *AtWSD1* could enhance drought tolerance of oil crop *Camelina sativa*, implying that cuticle biosynthesis genes identified from model plants could also be employed for the transgenic improvement of crop plants like wheat and barley (Lee et al., 2014; Abdullah et al., 2021). Due to biosafety concerns, selectable marker genes (SMGs) used for the selection of transformants should be eliminated from GM crops, which was facilitated by marker-free transgenic (MFT) strategies (Ling et al., 2016; Wang G. P. et al., 2016; Ahmad and Mukhtar, 2017; Wang et al., 2017). By employing the double right border (DRB) T-DNA vector, Cao et al. successfully constructed the marker-free and transgene insertion site-defined (MFTID) transgenic wheat plants for silencing *lipoxygenase* (*LOX*) gene (Cao et al., 2020). These MFTID transgenic wheat plants exhibited attenuated *LOX* gene expression, and improved grain storability, and fatty acid content, thereby paving a path for creating MFTID plants with altered cuticle traits in wheat and barley (**Figure 1**) (Cao et al., 2020). As recalcitrant crops, wheat and barley have low rates of transformation and regeneration (Mrízová et al., 2014; Hensel, 2020; Mirzaee et al., 2022; Wijerathna-Yapa et al., 2022). Over-expression of the WUSCHEL family gene *TaWOX5* and the chimeric gene harboring wheat *TaGRF4* and *TaGIF1* were reported to improve wheat efficiency of transformation and regeneration respectively (Debernardi et al., 2020; Wang et al., 2022). These breakthroughs in crop transformation and regeneration would certainly facilitate the genetic engineering of the cuticle biosynthesis genes in wheat and barley.

As an advanced genome editing (GE) technique, the CRISPR-Cas9 (clustered regularly interspaced short palindromic repeats-CRISPR associated 9) system has been extensively employed for functional genomics and trait improvement in model and crop plants (Chen et al., 2019; Manghwar et al., 2019; Zhu et al., 2020; Gao, 2021). Knockout of *MYS1* and *MYS2*, transcription repressors of *DECREASE WAX BIOSYNTHESIS* (*DEWAX*), by CRISPR-Cas9 system, resulted in the reduced wax loads and attenuated plant drought tolerance, suggesting that genome editing of cuticle biosynthesis genes could effectively alter plant stress tolerance (Liu et al., 2022c). However, the conventional application of the CRISPR-Cas9 system necessitates plant transformation and regeneration, which hinders its use in wheat and barley breeding. Interestingly, Li et al. established a tissue culture-free genome editing approach in Cas9-overexpressed (Cas9-OE) wheat plants by employing an engineered BSMV-based sgRNA (BSMV-sg) delivery vector (Li et al., 2021). By adding RNA mobility sequence in the virus vector, Chen et al. and Ellison et al. successfully enhanced the editing rate of this convenient virus-mediated gene editing system, which paved a new path for genetic manipulation of cuticle biosynthesis genes in wheat and barley (**Figure 1**) (Ellison et al., 2020; Chen H. et al., 2022).

In traditional mutation breeding, genetic mutations were induced by chemical, physical and biological agents such as ethyl methanesulfonate (EMS), X-rays, gamma rays, fast neutrons, and T-DNAs. Compared with genetic engineering and genome editing, this traditional mutation breeding based on random mutagenesis is labor-intensive, time-consuming, and less effective (Holme et al., 2019). As an advanced strategy in targeted mutation breeding, targeting induced local lesions (TILLING) deploys saturated mutagenesis and high-throughput screening approaches to efficiently introduce single-nucleotide mutation to any genomic regions like cuticle biosynthesis genes (McCallum et al., 2000; Kurowska et al., 2011; Chen et al., 2014). A *drought-insensitive TILLING line 1* (*ditl1*) mutant was recently identified from the rice TILLING mutant population and was revealed to harbor mutation in the cuticle biosynthesis-related WSD1-like gene (Choi et al., 2022). These induced mutations with desired cuticle traits could be introduced into elite cultivars of wheat and barley *via* cross breeding facilitated by advanced marker-assisted selection (MAS) approaches (**Figure 1**) (Hickey et al., 2019; Thudi et al, 2021).

Although the genetic manipulation of some cuticle biosynthesis genes could enhance abiotic and biotic stress tolerance in wheat and barley, many challenges need to be overcome regarding the exploitation of cuticle biosynthesis for wheat and barley improvement. For instance, present cuticle phenotyping techniques such as GC-MS (gas chromatography-mass spectrometry) and MALDI (matrix assisted laser desorption/ionization) imaging are low throughput and time-consuming, and high-throughput and high-precision methods needed to be developed for identifying wheat and barley mutants with cuticle traits (Petit et al., 2017). Furthermore, over-expression of cuticle biosynthesis genes usually enhances plant stress resilience with yield failure due to altered metabolic and energy flux. It is, therefore, vital for breeders to identify new cuticle biosynthesis genes conferring plant stress resilience without yield penalty. Moreover, strict policy regulations have been imposed on GMOs (genetically modified organisms) in some countries, and these regulations needed to be modified for placing wheat and barley varieties developed by genetic engineering and/or genome editing of cuticle biosynthesis genes into markets (Wolt and Wolf, 2018; Eriksson et al., 2020; Redden, 2021; Hundleby et al., 2022).

## Concluding remarks

Herein, we provide an overview of recent progress in the understanding of cuticle biosynthesis in wheat and barley, and highlight the contribution of cuticle biosynthesis in the adaptation of wheat and barley to environmental challenges. Current strategies and limitations on exploiting cuticle biosynthesis for wheat and barley improvement are discussed. As depicted in **Figure 1**, genetic manipulation of cuticle biosynthesis genes by transgenic breeding, genome editing, mutation breeding, and cross breeding could result in cuticle reinforcement and lead to improved performance of wheat and barley under stressful environments. Although the past decades have seen unprecedented proceedings in the exploration and exploitation of cuticle biosynthesis, we still have a long way to go toward fully understanding cuticle biosynthesis in wheat and barley. For instance, most of the characterized cuticle biosynthesis genes come from model plants, while wheat and barley genes involved in the biosynthesis of cuticular lipids, especially cutin monomers, are poorly understood. Furthermore, cuticle compositions of wheat and barley vary along with plant developmental stages and environmental conditions, but the response mechanism of cuticle biosynthesis to developmental and environmental cues remains to be uncovered in wheat and barley. Moreover, the cuticle plays a vital role in plant tolerance to abiotic stresses, but the functions and mechanisms of cuticle biosynthesis in the adaptation of wheat and barley to salinity, temperature, and UV stresses remain to be disclosed. In addition, wax signals from wheat and barley cuticles are revealed to facilitate conidial germination of powdery mildew, but whether and how cuticle biosynthesis regulates interactions of wheat and barley with other P&Ps such as bacterial pathogens and pests is still unknown. With advances in the understanding of cuticle biosynthetic machinery in wheat and barley, manipulating cuticle biosynthesis would certainly promote crop improvement for stress resilience and disease resistance.

## Author contributions

CC and XW wrote this manuscript. All authors have read and agreed to the published version of the manuscript.

## Funding

This work was funded by the Natural Science Foundation of Shandong Province (ZR2022MC008, ZR2017BC109), National Natural Science Foundation of China (31701412), the Qingdao Science and Technology Bureau Fund (17-1-1-50-jch) and Qingdao University Fund (DC1900005385).

## Conflict of interest

The authors declare that the research was conducted in the absence of any commercial or financial relationships that could be construed as a potential conflict of interest.

## Publisher’s note

All claims expressed in this article are solely those of the authors and do not necessarily represent those of their affiliated organizations, or those of the publisher, the editors and the reviewers. Any product that may be evaluated in this article, or claim that may be made by its manufacturer, is not guaranteed or endorsed by the publisher.
